# Development and Preliminary Feasibility of iByte4Health: A Mobile Health (mHealth) Pediatric Obesity Prevention Intervention to Engage Parents with Low-Income of Children 2–9 Years

**DOI:** 10.3390/nu13124240

**Published:** 2021-11-25

**Authors:** Gina L. Tripicchio, Melissa Kay, Sharon Herring, Travis Cos, Carolyn Bresnahan, Danielle Gartner, Laura Stout Sosinsky, Sarah B. Bass

**Affiliations:** 1 Center for Obesity Research and Education, Temple University, Philadelphia, 19140 PA, USA; sharon.herring@temple.edu; 2 Department of Social and Behavioral Sciences, Temple University, Philadelphia, 19122 PA, USA; sarah.bauerle.bass@temple.edu; 3 Department of Psychology & Neuroscience, Duke University, Durham, 27708 NC, USA; melissa.kay@duke.edu; 4 Department of Health and Rehabilitation Sciences, Temple University, Philadelphia, 19122 PA, USA; travis.cos@gmail.com; 5 Department of Health Policy and Management, Johns Hopkins University, Baltimore, 21205 MD, USA; cbresna5@jhmi.edu; 6 Health Science Center at Houston, University of Texas, Houston, 77030 TX, USA; danielle.j.gartner@uth.tmc.edu; 7 Research and Evaluation Group, Public Health Management Corporation, Philadelphia, 19102 PA, USA; lsosinsky@phmc.org

**Keywords:** pediatric obesity, mobile health, health disparities, obesity prevention, digital health

## Abstract

This research describes the development and preliminary feasibility of iByte4Health, a mobile health (mHealth) obesity prevention intervention designed for parents with a low-income of children 2–9 years of age. Study 1 (*n* = 36) presents findings from formative work used to develop the program. Study 2 (*n* = 23) presents a 2-week proof-of-concept feasibility testing of iByte4Health, including participant acceptability, utilization, and engagement. Based on Study 1, iByte4Health was designed as a text-messaging program, targeting barriers and challenges identified by parents of young children for six key obesity prevention behaviors: (1) snacking; (2) physical activity; (3) sleep; (4) sugary drinks; (5) fruit and vegetable intake; and (6) healthy cooking at home. In Study 2, participants demonstrated high program retention (95.7% at follow-up) and acceptability (90.9% reported liking or loving the program). Users were engaged with the program; 87.0% responded to at least one self-monitoring text message; 90.9% found the videos and linked content to be helpful or extremely helpful; 86.4% found text messages helpful or extremely helpful. iByte4Health is a community-informed, evidenced-based program that holds promise for obesity prevention efforts, especially for those families at the increased risk of obesity and related disparities. Future work is warranted to test the efficacy of the program.

## 1. Introduction

Pediatric obesity remains a pressing public health issue and disparities persist among racial and ethnic minority youth and those living in low-income households [1,2,3]. Innovative obesity prevention strategies are needed to address disparities and to improve the long-term health of children by preventing the onset of obesity and related co-morbidities including high-blood pressure and asthma [4,5]. Currently, obesity prevention interventions show a limited impact among populations at increased risk and trials that include socially marginalized populations often experience poor engagement and high attrition [6]. Technology, specifically mobile health (mHealth), provides an approach for addressing key barriers to obesity prevention including increasing access and allowing for simplified and engaging content to be sent directly to participants [7]. mHealth programs are cost-effective, scalable, and highly acceptable, but to date, few have been designed to keep pace with current advances in technology [8]. The use of smartphones to deliver mHealth programs holds promise for addressing obesity-related disparities among racial and ethnic minority families, given that populations who identify as Black and Hispanic are more likely to rely on smartphones to access the internet, rather than computers or home-based internet [9]. Therefore, there remains potential for developing engaging, dynamic, and community-informed mHealth programs that can be delivered via smartphone to address obesity disparities in high-risk pediatric populations.

To date, however, a majority of parent-focused digital health interventions aimed at shifting weight status in children have been ineffective [10]. Most mHealth intervention approaches have been limited to traditional short message service (SMS), apps and web-based platforms; few programs have been tailored to parent preferences or designed with content parents can share with their children [11,12,13]. Smartphones present exciting opportunities to create and send dynamic content to parents, like YouTube videos, picture messages, and linked resources. This versatility holds potential for increasing the amount of tailored intervention material that can be sent to parents and addressing barriers like digital literacy and website or app fatigue that may be experienced in interventions [12,14]. Maximizing participant engagement is an important feature associated with retention and behavior change in mHealth programs and is needed to enhance the relevance of health communication messages for targeted audiences [15]. Designing engaging programs is critical, as effective engagement is a key feature of behavior change and is associated with longer-term impacts [16]. Therefore, mHealth obesity prevention programs should focus on relevant content that promotes engagement and integrates evidence-based behavioral strategies.

The purpose of this paper is to present an overview of the development and preliminary testing of iByte4Health, a mHealth obesity prevention program delivered via smartphone that integrates parent and child-facing content to facilitate discussion around key health behaviors, and allows parents and children to work together towards goals, and engage in behavior change as a family. The goal of iByte4Health was to develop a low-cost, scalable, entirely virtual, and evidence-based prevention program that could be easily disseminated to reach families with children at-risk of obesity. Using best-practice health communication principles, we used an iterative approach to develop a theory-informed and engaging mHealth intervention [17]. Findings and insights from mixed-methods formative work (Study 1), and preliminary feasibility and acceptability data from a two-week proof-of-concept pilot study with parents (Study 2) are presented. Study 1 was conducted with 36 parents/caregivers of children 2–9 years of age from a Federally Qualified Health Center (FQHC), to determine preferences for the development of iByte4Health. The goal of the formative work was to better understand each of the following in the target population: (1) preferences for content focus and key barriers to improving health behaviors in children; (2) technology access; and (3) ideal digital program format and frequency. Study 2 was then subsequently conducted among 23 parents of children 2–9 years of age, using a two-week pilot version of the iByte4Health program to examine overall program feasibility, utilization, engagement, and acceptability.

## 2. Materials and Methods

### 2.1. Program Overview

iByte4Health is a mHealth obesity prevention program delivered via smartphone, which addresses key barriers to implementing obesity prevention strategies for families with children who may be at increased risk for obesity. Specifically, the program provides tailored content that is evidence-based and community-informed. A key feature of the program is the integration of multimedia content (e.g., videos, infographics, games) that facilitates discussion around key health behaviors and allows parents and children to work together towards goals and engage in behavior change as a family. The program also leverages technology to increase access and address barriers to recruitment, retention, engagement, and in-person attendance, which increases potential for addressing disparities among vulnerable groups. Lastly, the program integrates behavioral strategies like goal-setting and self-monitoring and provides interactive and personalized feedback to guide participants towards behavior change. The mHealth delivery platform also makes the program scalable, easy to disseminate, and allows for integration into clinical settings like primary care.

### 2.2. Study 1: Formative Development

In the first phase of formative work (June–August 2019), 36 parents and caregivers of children 2–9 years of age were recruited from a FQHC in North Philadelphia to provide insights, through in-depth interviews and questionnaires, on their primary health concerns for their child, technology use, preferences regarding an mHealth intervention (e.g., types of content, frequency of messages) and key barriers to addressing health behaviors in their children. Parents/caregivers completed in-person surveys that were administered by trained study staff and included qualitative and open-ended questions. Participants were asked about their concerns and desired support for several obesity prevention targets including sugary drinks, physical activity, fast food intake, screen time, snacking, sleep, and fruit and vegetable intake. These obesity prevention targets were adapted from the Family Nutrition and Physical Activity (FNPA) Screening tool, a validated behaviorally focused measure that assesses obesogenic influences for young children [18,19,20]. Additional behavioral targets, including questions on stress and picky eating, were included based on insights from behavioral health counselors and pediatric providers at the partner FQHCs regarding topics that commonly came up in counseling conversations with parents. Parents were also asked about technology access and preferences for receiving health information about their child via phone.

This formative phase tested a variety of content and messages that were designed to address potential barriers to digital literacy and to make content more appealing. Messages included traditional text-based messages and picture messages. The survey contained about 75 questions, including demographics, and took approximately 15 min to complete on a digital tablet. Parents were then asked probing questions to provide additional insights and feedback about their impressions and preferences for messages. The brief interview, which lasted 10–30 min, was recorded electronically, transcribed, and synthesized to identify key themes and provide context for quantitative responses. Participants who were interested in participating in the survey but did not want to be recorded were still included in the study. The study was conducted according to the guidelines of the Declaration of Helsinki and approved by the Institutional Review Board (or Ethics Committee) of Temple University (protocol 25684, 28 May 2019).

### 2.3. iByte4Health Program Development

Based on the formative work conducted with parents/caregivers, iByte4Health was designed as a parent-focused 6-week pilot mHealth text-messaging program that targets key health behaviors to prevent obesity in vulnerable children. The six obesity prevention targets were synthesized to align with the key content areas of parent interest and intended to cover one topic per week. The six topics include: (1) snacking, (2) physical activity, (3) sleep, (4) sugary drinks, (5) fruit and vegetable intake, and (6) healthy cooking at home. Barriers and challenges related to each of these key health behaviors including structure, feeding and mealtime practices, picky eating, and stress management were integrated within each of these overarching themes. The program was theoretically informed by social cognitive theory (SCT), seeking to influence parents’ knowledge, self-efficacy, and self-regulation (using goal-setting and self-monitoring) to directly influence children’s environment and routines [21]. Behavior change techniques (BCTs), which are defined by Michie et al., as a theory-linked taxonomy of components in interventions that are essential for promoting behavior change (e.g., self-monitoring, problem solving), were also used to guide intervention development [22,23]. The videos developed for iByte4Health were coded with key BCTs including: goals and planning, feedback and monitoring, shaping knowledge, natural consequences, repetition and substitution, comparison of outcomes, reward and threat, antecedents, identity, and self-belief [22]. Text-messages were designed to address four behavior change strategies that corresponded to SCT constructs: goal setting, role modeling, improving the home environment, and addressing barriers.

iByte4Health consists of four key components: (1) animated YouTube videos (<3 min each) that introduce the weekly themes and behavior change goals; (2) daily text-messages that provide strategies and tips for changing the weekly target behavior; (3) engaging supplemental content to provide additional support for each theme; and (4) twice weekly self-monitoring and feedback messages. Each week begins with a short, animated video that provides evidence-based information about the importance of the weekly target health behavior. Videos were created based on structured templates, which included age-specific evidence-based recommendations from the American Academy of Pediatrics, Bright Futures: Nutrition, the 2015–2020 Dietary Guidelines for Americans, and the Physical Activity Guidelines for Americans (2nd Edition) [24,25,26]. The videos also included information on the benefits of addressing the target behavior in children, common barriers (derived from formative work) and corresponding strategies on how to address them, realistic goals for improving the target behavior, and strategies for how to self-monitor progress towards the goal. Videos can be viewed on the iByte4Health YouTube channel (link in Supplementary Materials).

**Figure 1:**
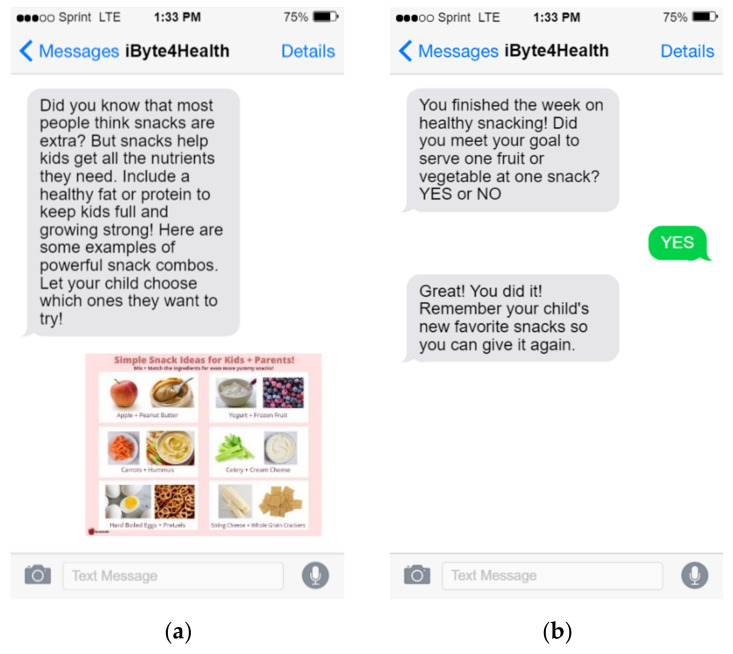
Screenshots of iByte4Health text message content. (**a**) Example of a daily messages with a visual guide; (**b**) Example of a summative self-monitoring text message at the end of the week and a tailored response.

**Figure 2:**
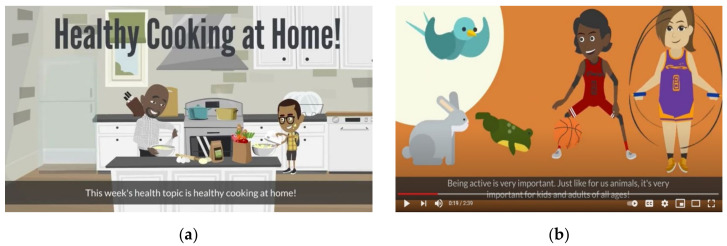
Screenshots of YouTube video content: (**a**) image from the Healthy Cooking at Home video; (**b**) image from the Physical activity video.

Short, easy to understand, daily messages were created to support the weekly theme and provided simple, clear guidance to help address common barriers and challenges reported by parents in formative work. Messages were sent once daily in the morning (at approximately 10 a.m.) Every message was accompanied by supplemental content that included infographics or links to more information. Infographics were sent as text message images and could be saved directly to participants’ devices. Most of the visual content was developed by the study team and included tips (e.g., healthy snack ideas), games (e.g., exercise bingo), and other visuals to support text-message targets (e.g., picture-based guide on how to structure snack time). Additionally, parents received two self-monitoring text-messages per week in the afternoon. Participants were asked to provide “YES” or “NO” responses to the self-monitoring messages and feedback was provided based on participant response. One message was sent mid-week to assess progress towards their weekly goal and to help troubleshoot challenges. If participants reported meeting their goal, they received a message to positively reinforce the behavior. If parents had not yet met the goal, they received a tip to help them achieve the goal before the end of the week. A final self-monitoring message was sent at the end of the week to assess goal attainment for the weekly target behavior before a new weekly theme was introduced. Examples of text-messages are presented in Figure 1 along with screenshots of the animated videos in Figure 2.

### 2.4. Study 2: Proof-of-Concept Feasibility Testing

Proof-of-concept testing was conducted to determine preliminary usability, acceptability, and engagement with iByte4Health. Measures are detailed below. Delays in study timelines and program development occurred due to the COVID-19 pandemic, and as a result an adapted two-week version of the program was created for initial proof-of-concept testing. The pilot study was conducted remotely during April–May 2021. Snacking and physical activity were selected as the two health behavior targets for this two-week study, given that parents/caregivers reported significant interest in these targets during formative work. Additionally, these two behaviors were also significantly impacted by COVID-19 and were thought to be most relevant for parents and youth during the proof-of-concept testing [27].

Participants were recruited through one of three methods: (1) referral from the integrated behavioral health counselor from two local FQHC partner clinics; (2) contacting families who participated in previous research at the center where the study was conducted and agreed to be contacted for future studies; or (3) friends and family referral. Parents completed an eligibility screener and consent form via a data management system, Research Electronic Data Capture (REDCap), a secure software program that is designed to capture data and support clinical and translational research projects [28]. To be eligible, participants had to be at least 18 years of age, a parent or legal guardian to at least one child 2–9 years of age, able to read, write in and understand English, have a working smartphone, and agree to send and receive text-messages for the two-week intervention. After completing eligibility and consent, the baseline survey was automatically generated via REDCap and upon completion participants were manually added to the iByte4Health program. After the two-week intervention, participants completed an end-of-program survey via REDCap. Each survey contained about 40 questions each and took approximately 15–20 min to complete. Participants received $40 for participation in the study. The study was conducted according to the guidelines of the Declaration of Helsinki and was approved by the Institutional Review Board (or Ethics Committee) at Temple University (protocol #28159, 9 March 2021). Informed consent was electronically obtained from all subjects involved in the study.

Program acceptability was assessed using responses to a series of five-point Likert scale questions for the overall program (1 = I did not like it at all, 2 = I didn’t really like it, 3 = I somewhat liked it, 4 = I liked it, 5 = I loved it!), and individual program components. For example, participants were asked to state how much they agreed or disagreed with the following statements about the overall program (1 = Strongly disagree, to 5 =Strongly agree): “The overall program was engaging”, “I understood the messages and content in the program”, “The program is relevant for me and my family.” Questions also assessed how helpful participants found each part of the program (1 = Not at all helpful, 5 = Extremely helpful) including: “How helpful or useful were the text messages received during the program?” “How useful or helpful were the videos received during the program?” and “How useful or helpful was the linked content that was provided with the text messages?” For each program component, program utilization was assessed by asking participants how much they viewed (1 = All of it, 2 = Most of it, 3 = Some of it or 4 = None of it) and how they would rate the amount of content that was provided (1 = Too much, 2 = Just right, 3 = Not enough). Participants were also asked if they set new goals as part of the program (yes/no) and if they were able to achieve those goals (yes/no). Program engagement was assessed through text-message delivery confirmation on the carrier platform and by examining participant responses to self-monitoring text messages. Descriptive statistics were used to analyze baseline characteristics and responses to program utilization, engagement, and acceptability questions.

A modified version of the 20-item FNPA was used to examine preliminary efficacy in parenting behaviors related to the intervention. Four questions, which were not directly related to the behavioral targets, were removed to reduce the participant burden. The survey was administered at baseline and follow-up. However, given the limited sample size, no statistical tests were used to examine pre-post differences and only descriptive results are presented for questions that assessed the key targets of the intervention, specifically, parent responses to the following four questions assessing snacking behaviors: “How often does your child eat fruits and vegetables at meals or snacks?”, “How often does your family monitor the amount of candy, chips and cookies your child eats?”, “How often does your family use candy, ice cream or other foods as a reward for good behavior?” and “How often does your child eat while watching TV?” These three questions were used to examine physical activity: “How often does your family encourage your child to be physically active?”, “How often does your child do physical activities with at least one other family member?”, “How often does your child do something physically active when he/she has free time?” Responses were provided using a 4-point Likert scale (1 = Never, 2 = Sometimes, 3 = Often, 4 = Always). SPSS Version 25 was used to calculate means, SDs, and percentages.

## 3. Results

### 3.1. Study 1: Formative Development

#### 3.1.1. Participant Demographics

Of the 36 participants in the formative study, 69.4% identified as African American/Black, 13.9% White, and 19.4% other (including mixed race). More than one-quarter identified as Hispanic (30.6%). The mean caregiver age was 33.5 years (SD = 10.5) and mean child age was 5.4 years (SD = 2.9). Approximately half (54.0%) of caregivers had a high school level education or less and on average participants had 3.0 (SD = 1.9) children living in their household. Many participants were mothers (58.3%), but fathers (22.2%) and other caregivers (including grandparents and stepparents) also participated (19.5%).

#### 3.1.2. Preferences for Content Focus and Key Barriers to Improving Health in Children

Participants were interested in receiving information across a range of targets related to their child’s health, but a primary concern was their own feeding practices. Most parents/caregivers wanted tips on how to feed children (84.2%). Respondents provided additional insights on their challenges around feeding children which included mealtime practices (e.g., getting children to sit at the table), beverage selection (e.g., how to get children to drink less juice and more water), picky eating (e.g., how to get children to eat fruits and vegetables), screen time (especially during meals), and structure (e.g., how many snacks per day, best types of food combinations for different meals). Snacking habits (73.3%), stress (71.0%), physical activity (71.0%), and establishing family meals (71.0%) were also content areas of interest for most respondents. Parents/caregivers were also eager to get more information about how to improve the healthfulness of snacks. For example, one parent said, “If he sees junk food that’s all he wants- he doesn’t want the healthier snacks…so maybe tips on ways to make healthier snacks more appealing.” Parents also expressed challenges with sleep- “[their child] throws a fit when it’s bedtime and needs to learn routine”, and screen time- “Screen time is a big thing and trying to get a phone away from him is hard”. Parents were also generally concerned about stress for themselves and their child, with one parent saying, “Children deal with anxiety… and stress can affect parents.”

#### 3.1.3. Technology Access

When assessing technology access and preferences, 94.7% of parents/caregivers reported having a working cell phone, 84.0% had unlimited messaging and data plans, and 88.9% reported using their phones to access the internet. Most parents (78.4%) were interested in receiving text messages with health information about their child.

#### 3.1.4. Digital Program Format and Frequency

Parents/caregivers provided significant insights on preferences for enhancing the digital program structure. When asked about messages, parents/caregivers reported liking both picture and text-based messages. Parents/caregivers shared that the picture messages were more engaging and could easily be shared with their children while the text-based messages were important for getting clear and detailed information. Most parents/caregivers (90%) expressed wanting additional content (e.g., videos, links to websites) and all parents wanted content that would promote conversation with their child. They liked the idea of setting goals and expressed that content should be clear and concise, easy to understand, and engaging. They also liked the idea of receiving pictures or content that they could save to their phone and refer to at a later time. When asked about their preference for frequency of message delivery, more than half of respondents said they would prefer one or more messages per day (53.2%). A little over half (51.7%) said they preferred messages be sent in the morning and 34.4% had no preference on timing. Parents/caregivers indicated that the consistent delivery of messages (e.g., daily in the morning) would create a structure and help them prepare for the day or the week.

### 3.2. Study 2: Proof-of-Concept Feasibility Testing

#### 3.2.1. Participant Demographics

A total of 23 parents (e.g., mother or father) were recruited for the two-week pilot intervention. Parents were 95.7% female, 34.4 years (SD = 7.9), 65.2% identified as African American/Black, and 13.0% identified as Hispanic/Latinx. Most (65.2%) reported use of at least one form of government assistance, 39.1% had a high school education or less, 26.1% reported some college, and 30.3% were college graduates or higher. About half (47.8%) reported 1–2 children living in their household while 52.1% reported three or more children. Children were on average 6.6 years (SD = 2.0).

#### 3.2.2. Program Utilization and Engagement

All (100%) of the text-messages were successfully received by participants (according to carrier records on the text-message server) and all but one (95.7%) of the parents completed follow-up at the end of the two-week study. Responses to self-monitoring text messages were high with 87.0% responding to at least one of the four self-monitoring prompts. Responses to self-monitoring texts were higher at the end of the week (average response rate 80.4%) compared to the mid-week messages (average response rate 58.7%). In addition to engagement with self-monitoring texts, parents demonstrated other forms of unprompted engagement with the program including “liking” or “loving” text-messages as well as sending pictures of what children were eating and asking questions. The principal investigator responded to all participant questions within 24 h of receiving questions via text-message.

Almost all parents (95.4%) reported reading all or most of the text-messages, 81.8% reported watching all or most of the videos, and 86.4% reported viewing all or most of the linked content. Most parents (81.8%) reported sharing the program content with their child and 66.6% reported that their children liked or loved the content while 27.8% of children felt neutral about it.

#### 3.2.3. Program Acceptability

Almost all parents (90.9%) reported liking or loving the iByte4Health program. Most parents (90.9%) reported setting new goals as part of the program and 87.0% reported achieving their goals. Overall, parents agreed or strongly agreed that the program was engaging (90.9%), that they understood the messages and content (90.9%), that the program was relevant for them and their families (86.4%), and that the program was helpful (86.4%). When asked about how helpful the program components were, parents reported the following to be helpful or extremely helpful: videos (90.9%), linked content (90.9%), and text messages (86.4%). All parents (100%) who responded to the follow-up agreed that the program was helpful for providing them with information to keep their children healthy.

#### 3.2.4. Program Content and Dose

Overall, parents reported that the number of text-messages received during the program (e.g., daily messages with two additional monitoring messages) was just right (95.5%), with only one parent saying it was not enough (4.5%). Most parents (90.9%) also said the amount of linked content was just right. In contrast, while a majority of parents (77.3%) reported that the number of videos received was just right (one per week), 13.6% said they wanted more videos, and several parents reiterated this point again in the open-ended feedback.

#### 3.2.5. Behavioral Targets

When examining parent-feeding practices, parents reported a decrease in how often their child ate meals and snacks in front of the TV; 52.2% reported doing this often/always at baseline compared to 34.7% at follow-up. Parents reported increases in monitoring child intake of snacks; 69.5% reported doing this often/always at baseline compared to 77.3% at follow-up. Most interestingly, parents reported reducing the use of snack foods as a reward; 69.5% of parents reported doing this often/always at baseline while no parents (0%) reported doing this often/always at follow-up (31.8% reported never doing it and 68.2% reported doing this sometimes).

When examining parenting practices around child physical activity, there was a slight increase in parent reported encouragement of child physical activity; 82.6% reported doing this often/always at baseline compared to 86.4% at follow-up. There was also a slight increase in the amount of physical activity that children engage in with family members; 65.2% reported doing this often/always at baseline compared to 68.2% at follow-up. Lastly, parents reported an increase in the amount of physical activity children do when they have free time; 73.9% reported this occurring often/always at baseline compared to 81.9% at follow-up.

## 4. Discussion

iByte4Health is a community-informed, evidence-based mHealth intervention designed to address barriers and challenges to obesogenic behaviors in families with young children 2–9 years from underserved populations who may be at-risk of obesity. Using an iterative evaluation strategy, results suggest that this program is feasible and acceptable to this target population and ensured input from the target population at each development stage. Almost all parents said that the program content was relevant to them and their families, highlighting the critical importance of formative work to develop the tailored content delivered in mHealth interventions. Overall, engagement in the iByte4Helath pilot program was high and program dose and content was highly rated among pilot users. Parents also used features of their smartphone like “loving” or “liking” a message sent from iByte4Health, and even though this was unprompted, it served as a quick but effective way to learn participant preferences without being burdensome. Preliminary findings suggest that text messaging is a feasible and interactive way for parents and caregivers in at-risk groups to engage in health communication and obesity prevention programming.

Participants reported that daily messages (and sometimes twice daily) were not too burdensome and the video and other picture-based content was highly rated. Participants even requested additional child-facing content like YouTube videos. Parents found the additional resources helpful and relevant to improving the health of their children. Most parents shared the program content with their children, suggesting that the content was not only relevant and interactive for the parent but also served as a means of communicating with their child about ways to improve their health. This study also confirmed that smartphones are a feasible and effective way to leverage mHealth’s potential for engaging individuals living in low-income households in health education, which can lead to the adoption of health-promoting behaviors [29].

A primary goal of this study was to develop an obesity prevention program that addresses current barriers in lack of access to obesity prevention programs for high-risk groups. This program can be readily and easily accessed by patients at any time via smartphones and is designed to enhance engagement and relevance. Additionally, this program can be scaled to provide a cost-effective approach for reaching larger audiences. In this pilot iteration, if participants sent questions or comments, they received individual responses from the principal investigator. However, text-messages could be automated if the intervention is scaled up. Additionally, individual responses could be managed by study staff or clinical teams and still be less burdensome and require fewer resources than other behavioral intervention approaches like traditional in-person sessions.

While this study has strengths, there are also limitations to consider when interpreting findings from this work. First, study timelines and evaluation plans were significantly disrupted due to COVID-19. This extended the amount of time between Study 1 and Study 2, delayed contracts and IRB approvals, and precluded the ability to recruit in-person for Study 2 or conduct objective measures. Additionally, both studies used small, convenience samples limiting the generalizability of findings. Study 1 could have been influenced by response bias since participants responded to open-ended questions asked by the research team and Study 2 used a pre-post design and was not powered to detect meaningful differences in outcomes. Finally, given that Study 2 was only conducted for two weeks, it remains unknown if participants would stay engaged in the program long-term. Future studies should examine what level of engagement is required to achieve behavior change and determine the optimal length of the program.

Partnerships with two FQHCs supported the development of this project. For future clinical integration, this program can serve as a referral program for providers to address current limitations to delivering obesity prevention care in the primary care setting. Primary care providers can screen for obesity risk as part of standard care, and then refer to behavioral health counselors who can assess familial needs and connect interested parents and caregivers with iByte4Health. To determine optimal implementation for this approach, further work is needed. In addition to examining the program impact on parenting and child weight-related behaviors, it will be important to examine whether this program is best utilized as an adjunct to care or as a standalone program.

Finally, while all mHealth tools should use formative approaches to ensure content addresses the needs of the target audience, using a “person-centered” approach that highlights the need to understand the perspectives of the people who will use the tool is critical to the development of interventions that are salient and useful to the audience. This is a key component of whether the target audience will use, and continue using, a technology-based intervention [30]. Targeted content that is born out of formative work is more relevant and holds greater potential for impact than tools that include general information. Though simplistic, utilizing a common platform like text-messages holds more promise for long term impact because previous studies have shown that participants often lose interest with other mHealth platforms like apps [31,32].

## 5. Conclusions

In conclusion, this paper summarizes formative work conducted with parents in low-income communities with children who may be at risk for obesity, to determine preferences for an obesity prevention mHealth program and the preliminary feasibility of implementing this program. Key factors and barriers that influenced child health behaviors as well technology utilization and preferences were identified and guided the development of a comprehensive mHealth program delivered via text-message on smartphones. The iByte4Health program was tested using an initial proof-of-concept two-week pilot study and initial acceptability, satisfaction, and engagement were high. Given the findings, iByte4Health holds promise for engagement, scalability, and dissemination, especially for families at increased risk for obesity and related disparities. Next steps include testing the full 6-week program using a rigorous study design and integrating customization approaches to ensure that the program is responsive to personalized preferences and the unique needs of families.

## Data Availability

The data presented in this study are available on request from the corresponding author.

## Supplementary Materials

The following are available online, Video S1: iByte4Health YouTube Channel: https://www.youtube.com/channel/UC5Eovivb19o4hJm4zfNOoIA/videos.
